# Trans person: discrimination and barriers associated with health care

**DOI:** 10.1192/j.eurpsy.2023.2424

**Published:** 2023-07-19

**Authors:** I. Ferreira, A. Valente, A. Morais, R. Lopes

**Affiliations:** 1 Serviço de Pediatria Médica, Hospital Dona Estefânia, Lisboa; 2 Unidade Cuidados Continuados da Santa Casa da Misericórdia de Cinfães, Cinfães; 3 Serviço de Cirurgia Geral, Instituto Português de Oncologia, Lisboa; 4 UCP Enfermagem de Saúde Mental e Psiquiátrica, Escola Superior de Enfermagem de Coimbra, Coimbra, Portugal

## Abstract

**Introduction:**

Discrimination is seen as a behavioral response, caused by negative attitudes towards specific values of certain people, and can be considered an effective form of stigma.

The trans population has been the target of various forms of discrimination, not only through disrespect for their name and gender-appropriate pronouns, but also resorting to aggression, isolation, marginalization and consequent economic hardship. These experiences can negatively influence mental health (MH), namely a higher prevalence of substance use, depressive episodes, anxiety disorders and suicide attempts.

**Objectives:**

To identify the impact of discrimination and to determine the barriers associated with healthcare for trans person.

**Methods:**

The P[I][C]OD methodology was used to prepare the research question: How does discrimination create barriers to health care for transgender people?. Research carried out through the EBSCOhost search engine, in the CINAHL Complete, MEDLINE Complete and Academic Search Complete databases, using the MeSH descriptors: “transgender”, “health”, “barriers” and “discrimination”, conjugated to the Boolean AND and OR, the search expression was obtained: (TI transgender OR AB transgerder) AND (TI discrimination OR AB discrimination) AND (TI health OR AB health) AND (TI barriers OR AB barriers). Inclusion criteria: studies published from 2018-2022; available in full text; English, Portuguese or Spanish languages. Sample of 6 articles**Results:** Studies show that the trans person is often stigmatized, a victim of prejudice and discrimination in accessing health care, with an impact on MH. Barriers to accessing health care highlighted are: denial of care, lack of specialized services, lack of knowledge and support, failures in training and knowledge of guidelines by health professionals.

Prejudiced/insulting language and exclusion are episodes reported with high frequency. Denial and discouragement of exploration of gender identity are reported as episodes of indirect discrimination.

With regard to MH, the stress of gender minorities caused by stigma, prejudice and discrimination in health services creates a hostile and stressful social environment in the trans population, which causes MH problems.

**Conclusions:**

Prescription and barriers lead trans person to avoid accessing care, with short and long-term adverse health warnings. The absence and/or lack of knowledge of health professionals contribute to this problem, making it crucial to invest in their academic training and continuous training, as well as in clinical practice guidelines and guidance, with the aim of training professionals.

It is necessary to acquire knowledge about the health of the trans person, particularly about the specific health needs and the creation of an inclusive environment between professionals and the person being cared for. Increased support, knowledge about issues related to the trans population and access to care improve MH.

**Disclosure of Interest:**

None Declared

